# The role of aspirin in post-polypectomy bleeding – a retrospective survey

**DOI:** 10.1186/1471-230X-12-138

**Published:** 2012-10-10

**Authors:** Antony Pan, Martin Schlup, Ralf Lubcke, Annie Chou, Michael Schultz

**Affiliations:** 1 Gastroenterology Unit, Southern District Health Board, Dunedin, New Zealand; 2 Department of Medical and Surgical Sciences, Dunedin School of Medicine, University of Otago, Dunedin, New Zealand

**Keywords:** Post polypectomy bleeding, Aspirin

## Abstract

**Background:**

Bleeding following colonoscopic polypectomy is a common complication and has been reported to occur in up to 6.1% of patients. Several risk factors have been discussed but their overall contribution to post-polypectomy bleeding remains controversial. The aim of the study was to determine the rate of post polypectomy bleeding and to analyse the role of potential risk factors especially the role of aspirin.

**Methods:**

We conducted a retrospective cohort study of all patients who underwent polypectomy at Dunedin Hospital, New Zealand between January 2007 and June 2009.

**Results:**

During the study period, 514 patients underwent colonoscopy with polypectomy and a total of 1502 polyps were removed. From further analysis we excluded 21 patients; 15 patients had surgery immediately after colonoscopy for the diagnosis of colorectal carcinoma and 6 patients presented with symptoms of an acute lower gastrointestinal bleed prior to colonoscopy. Of the remaining 493 patients, 11 patients (2.2%) presented with post-polypectomy bleeding within 30 days of the investigation of which 8 were on aspirin. In total 145 patients were taking aspirin prior to colonoscopy and 348 patients were not taking aspirin. The use of aspirin was associated with an increased prevalence of post-polypectomy bleeding (OR=6.72, 95% C.I. 1.76 to 25.7). Interestingly, the use of non-steroidal anti-inflammatory drugs (NSAIDs) was not associated with risk of bleeding after polypectomy (OR=2.82, 95% C.I, 0.34 to 23.3).

**Conclusion:**

Our study confirmed a significantly increased risk of lower gastrointestinal bleeding following polypectomy in patients taking aspirin. We would recommend approaching the patient on aspirin coming forward for a colonoscopy with potential polypectomy with caution.

## Background

Colonoscopic colon cancer screening programs are being adopted world-wide. As a consequence, more patients undergo colonoscopy with polypectomy. Bleeding is a common complication that has been reported in approximately 0.2 – 6.1% of patients undergoing endoscopic polypectomy [1-3].

Aspirin is widely used for secondary prevention of myocardial infarction, stroke, and vascular death among patients who have survived an initial occlusive cardiovascular disease event [4-6]. In the UK, aspirin is used by 67% of the general population older than 35 years of age following a cardiovascular event but also by 7% in those without a cardiovascular event [5]. In New Zealand, at least 54% of the elderly population in residential care is using aspirin for secondary cardiovascular risk reduction [6].This has raised a management issue for patients on aspirin undergoing diagnostic and therapeutic endoscopy, as aspirin can prolong bleeding time after endoscopic biopsy and polypectomy [7,8]. The most recent European Society of Gastrointestinal Endoscopy (ESGE) Guideline published in 2011 recommends that aspirin does not need to be discontinued even before planned polypectomy [9]. The aim of our study was to retrospectively determine the rate of bleeding following colonoscopic polypectomy and to analyse the role of risk factors especially aspirin in this setting.

## Methods

### Study Population

Dunedin Hospital is the only secondary/tertiary hospital in the region, providing colonoscopic services to a population of approximately 193,803 [10]. Consecutive patients who underwent colonoscopic polypectomy between January 2007 and June 2009 at Dunedin Hospital, Dunedin, New Zealand were identified retrospectively from the endoscopy reporting database (EndoSmart, Dunedin, New Zealand). Retrospectively, each patients’ colonoscopy report was reviewed for polyp characteristics (sessile, semi-sessile, on a stalk), polyp size, method of polypectomy (cautery snare, hot or cold forceps), and number of polyps removed was recorded. The patients’ medical records were reviewed for demographical data, the indications for colonoscopy, use of NSAIDs, anticoagulant or antiplatelet agents and the prevalence of bleeding following polypectomy. A retrospective review of each patient’s clinical records, hospital files and electronic records was performed to establish the use of medications both prior to and post procedure. These records include general practitioners referral letter, nurse notes, and physicians’ inpatient and outpatient record.

Patients on anticoagulants follow a standardized protocol in our unit. Patients with current warfarin use for valvular heart disease are switched to enoxaparin 5 days prior colonoscopy and instructed to omit the enoxaparin dose on the morning of the procedure. Patients on warfarin for atrial fibrillation are instructed to stop warfarin 5 days prior procedure. Patient with elevated international normalized ratio (INR) greater than 1.5 are rescheduled. Patients on intravenous heparin are instructed to stop infusion 6 hours prior the procedure. Patients taking aspirin and NSAIDs are not told to discontinue the medication prior to procedure.

Patients’ medical records were used to identify any clinically important delayed post-polypectomy bleeding requiring attendance to hospital. Clinically important delayed post-polypectomy bleeding was defined as lower GI bleeding requiring transfusion, hospitalization, re-intervention or surgery within 30 days of the procedure. Patients who developed mild and self-limiting hematochezia after polypectomy and not requiring re-admission were not included. Dunedin Hospital is the only secondary/tertiary hospital in the region, and all regional hospitals share the same computer inpatient management system. Therefore, all patients who developed significant post-polypectomy bleeding requiring hospital admission were included in the study.

**Figure 1:**
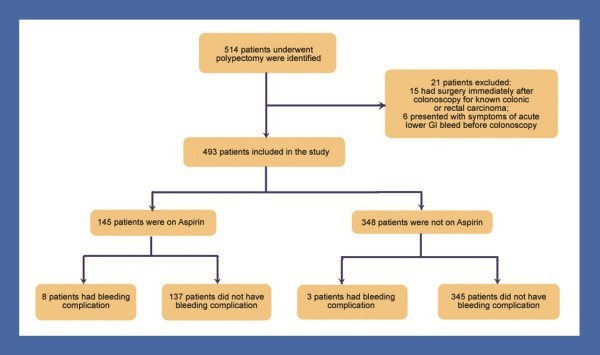
Methodology flow chart.

Patients were excluded if the procedure was performed primarily for acute upper or lower gastrointestinal bleeding. Patients who underwent surgery within 30 days after the procedure for reasons other than bleeding (e.g. colorectal carcinoma) were also excluded (Figure 1).

Statistical significance was assessed by determining the odds ratio (OR) from a conditional logistic model for matched pairs and 95% confidence interval (CI) around the OR were obtained as margins of error. The study is approved by Lower South Regional Ethics Committee, New Zealand.

## Results

A retrospective cohort study was conducted on all patients who underwent endoscopic polypectomy at Dunedin Hospital, New Zealand, between January 2007 and June 2009. During the study period, a total of over 1500 colonoscopies were performed. 514 patients underwent colonoscopy with polypectomy of which 21 patients were excluded (Figure 1). Of these, 15 patients had surgery immediately after colonoscopy for colonic or rectal carcinoma and 6 patients had presented with symptoms of acute lower GI bleed before colonoscopy. The most common indications for colonoscopy were iron-deficiency anemia, hematochezia, positive faecal occult blood, history of polyps, screening for colorectal cancer, and changing bowel habit.

A total of 1502 polyps were removed from the remaining 493 patients, 314 (63.4%) colonoscopies were for symptom assessment and 181 (36.6%) were for surveillance and screening. 145 patients were taking aspirin prior to colonoscopy and 348 patients were not taking aspirin.

Our two study populations (aspirin vs non aspirin) were significantly different (p<0.05) regarding the average age (Table 1) but there were no significant differences in gender, concomitant NSAID and dipyridamole use between these two groups.

**Table 1:** Characteristics of study patients

	**Aspirin group**	**Non-Aspirin group**	***p *value**
Number of patients	145	348	
Age			
Mean (SE)	72.1 (0.7)	62.2 (0.7)	p<0.05
Gender			
Male	83 (57.2%)	191 (54.9%)	NS, p=0.63
Concomitant NSAID use	4 (2.8%)	12 (3.5%)	NS, p=0.68
Concomitant dipyridamole use	2 (1.4%)	3 (0.9%)	NS, p=0.64
Polyp Data			
Total number of polypectomies	450	1052	
Range of number of polyps removed per Patient	1-30	1-26	
Number of polypectomies per patient (SE)	3.1 (0.3)	3.1 (0.5)	NS, p=0.79
Range of the size of polyp removed	2-40 mm	2-60mm	
Average size of largest polyp per patient (SE)	9.6 (0.7) mm	9.2 (0.5) mm	NS, p=0.59
Polypectomy technique			
Cold biopsy	35 (24.1%)	90 (25.9%)	NS, p=0.69
Hot biopsy	18 (12.4%)	51 (14.7%)	NS, p=0.50
Snare (blended current)	92 (63.4%)	207 (59.5%)	NS, p=0.43
Number of patients presented with post-polypectomy bleed	8 (5.5%)	3 (0.8%)	p<0.05

450 polypectomies were performed in the aspirin group and a total of 1052 in the non-aspirin group. The greatest number of polyps removed in a patient on aspirin was 30; the largest polyp removed was 40 mm. In the non-aspirin group the greatest number of polyps removed in a patient was 26; the largest was 60mm. There were no significant differences between two groups in regards to number of polypectomies per patient and average of polyp size per patient. The majority of polyps were removed by cautery snare using blended current with 63.4% in the aspirin group and 59.5% in the non-aspirin group. There was no difference between the two groups for use of different polypectomy techniques.

Eleven patients (2.2%) presented with post-polypectomy bleeding within 30 days post the procedure. Eight of the 11 patients were taking aspirin prior to colonoscopy compared to three patients who were not taking aspirin (Table 1). The use of aspirin was associated with a significantly increased prevalence of post-polypectomy bleeding (OR=6.72, 95% C.I. 1.76 to 25.7). The average age of patients in the aspirin group who experienced post-polypectomy bleeding was 72.1 compares to 62.2 in the non-aspirin group, however, there was no statistical significant difference between two groups. There was also no difference between the two groups regarding average polyp size (p=0.66), average number of polyps removed per patient (p=0.62), gender (p= 0.35), polypectomy technique (p=0.17) and concomitant use of NSAIDs and/or dipyridamole. The average days of admission for patients on aspirin presenting with post-polypectomy bleeding was 6.5 days compared to 1.6 days in patients not on aspirin. In the aspirin group, one patient required multiple transfusions, emergency surgery and long (22 days) hospitalisation. There was one death directly related to post-polypectomy bleeding. Of the remaining 6 patients, 5 required blood transfusions and one patient was treated conservatively. In the non-aspirin group, 2 patients were managed conservatively (observation only) and 1 patient required endoscopic therapy. There was no death in the non-aspirin group (Table 2).

**Table 2:** Characteristics of patients with post-polypectomy bleeding

	**Patients on aspirin (N=8)**	**Patients not on aspirin (N=3)**
Polypectomy technique		
Cautery snare (blended current)	6	3
Hot biopsy	2	0
Preventive clips placement	0	1
Preventive adrenalin injection	1	0
Average length of hospitalization (range)	6.5 (1-22) days	1.6 (1-2) days
Observation	1	2
Transfusion	7	0
Repeat colonoscopic intervention	0	1
Surgery	1	0
Death	1	0

A further 16 patients were taking NSAIDs prior to colonoscopy of which one presented with post-polypectomy bleeding. In contrast to aspirin, the use of NSAIDs was not associated with an increased risk of bleeding after polypectomy (OR=2.82, 95% C.I, 0.34 to 23.3).

## Discussion

Post-polypectomy bleeding (PPB) is a common complication that has been reported in up to 6.1% of patients [1-3]. Although in general, the prognosis for PPB is favourable, management of these patients can be costly, including hospitalization, blood transfusion and possible further endoscopic intervention. A small proportion of patients will require surgery or angiographic haemostasis.

Aspirin is widely used as secondary prophylaxis for cardiovascular events and has been shown to prolong colonic bleeding time after endoscopic biopsy and polypectomy [7,8]. The safety of polypectomy in patients taking aspirin is controversial. Our study retrospectively analysed post-polypectomy bleeding events in 493 patients and 1502 polypectomies. The use of aspirin was associated with a significantly increased risk of post-polypectomy bleeding (OR=6.72, 95% C.I. 1.76 to 25.7) and the average length of hospitalisation in patient with bleeding was significant longer in the aspirin group. No other factors including the use of NSAIDs prior to colonoscopy, polyp characteristics and method of polypectomy were associated with an increased risk of post-polypectomy bleeding.

A review of published studies on the use of aspirin in patients undergoing polypectomy demonstrates a wide variation. A published survey of members of the American Society of Gastrointestinal Endoscopy (ASGE) revealed that 81% of responders would consider discontinuation of aspirin before colonoscopy and 61% would not perform snare polypectomy in patients on aspirin [11]. In 1994, Shiffman et al. published a prospective study of 694 patients and found a small (<1%) increase in the risk of post-polypectomy bleeding in patients taking aspirin or NSAIDs [12]. Hui et al. [13] published a retrospective study involving 1657 patients who underwent colonoscopy with polypectomy, and concluded that the use of aspirin and NSAIDs was not associated with a higher risk of polypectomy-associated bleeding. A case-controlled study published by Yousfi et al. [14] also concluded that polypectomy can be safely performed in patients taking aspirin. Based on these studies a number of guidelines have been published, the ASGE guideline (2009) on the management of anticoagulation and antiplatelet therapy for endoscopic procedures suggests that aspirin and other NSAIDs in standard dosages do not increase the risk of significant bleeding after gastroscopy with biopsy, colonoscopy with biopsy, polypectomy or biliary sphincterotomy. The guideline recommends further that aspirin may be continued for all endoscopic procedures. However, when high risk procedures, including polypectomy, are planned, the individual clinician may elect to discontinue aspirin for 5 to 7 days before the procedure depending on the underlying indication for antiplatelet therapy [15]. This recommendation is in contrast to the recent European Society of Gastrointestinal Endoscopy (ESGE) guideline which recommends that aspirin does not need to be discontinued even before planned polypectomy irrespective of the polyp size [8]. A published survey in 2008 showed significant difference between Eastern and Western endoscopists in the management of antiplatelet medications before endoscopic procedure [16]. The Japanese Gastroenterological Endoscopy Society guideline recommends stopping aspirin before any endoscopic procedure, even if the procedure is considered low risk [16,17].

The use of aspirin for primary prevention remains controversial. Aspirin did not reduce the occurrence of stroke or “all cardiovascular events” compared to placebo in primary prevention patients with elevated blood pressure and no prior cardiovascular disease [5]. The Cochrane collaboration group concluded that the magnitude of harm is similar to the benefit, therefore aspirin cannot be recommended for primary prevention in patients with elevated blood pressure [18]. However, aspirin is established as an effective agent for secondary prevention in patients with proven cardiovascular disease [4]. Study [19] showed coronary events occurring after aspirin withdrawal represents 13.3% of coronary recurrence. Temporary withdrawal of oral antiplatelet agents is associated with increased risk of 30-days myocardial infarction and death [20]. Most of the patients with known heart disease are over 50 years of age and, therefore, in the group of patients where colonoscopy is required as screening and diagnostic procedure. We recommend approaching the patient who is on aspirin as secondary prevention coming forward for a colonoscopy with potential polypectomy with caution.

Our study has a number of limitations, most notably its retrospective nature and single center design. Our patients are not routinely contacted by our department after colonoscopy unless there is abnormal finding on the colonoscopy and therefore minor bleeding following polypectomy, not requiring hospital attendance was not captured. Our center shares the same electronic inpatient management system with regional hospitals, therefore all patients who presented with significant delayed post-polypectomy bleeding requiring hospitalization were identified for this study. Another possible limitation is the reliability of the recall history of aspirin use. To minimize this bias all available clinical records including pre- and post- procedural clinical letters, colonoscopy reports, nursing records and GP referral letter were checked to ensure the accuracy of medication use. We did not explore the temporal association and dosage of aspirin exposure with the risk of bleeding. Nor did we take the possibility of comorbid medical conditions which may increase the risk for post-polypectomy bleeding into account [21,22]. The average age of patients in aspirin group was significantly higher than for patients in the non-aspirin group. A possibly higher number of comorbidities might have contributed to the higher rate of post-polypectomy bleeding in the aspirin group. Other studies have identified advanced age as a significant predisposing factor for post-polyepectomy bleeding [23,24].

## Conclusion

In summary according to our results, the use of aspirin prior to colonoscopy is associated with a significantly increased risk of lower gastrointestinal bleeding following endoscopic polypectomy. Based on the limited benefit of aspirin as primary prevention we recommend to stop aspirin 5 to 7 days prior to planned polypectomy. Most of the patients with known heart disease are over 50 years of age and therefore in the group of patients where colonoscopy is required as a screening and diagnostic procedure. We recommend approaching the patient who is on aspirin as secondary prevention coming forward for a colonoscopy with potential polypectomy with caution.

## Competing interests

The authors declare no competing interests.

## Authors’ contribution

AP was involved in the design of the study, review of literature and writing of the manuscript. MS and RL participated in the design of the study. AC assisted in data collection, MS, corresponding author, was supervising the study, helped with the literature review and assisted in the writing and review of the manuscript. All authors read and approved the manuscript.

## Pre-publication history

The pre-publication history for this paper can be accessed here:

http://www.biomedcentral.com/1471-230X/12/138/prepub
